# Supplementary material for: Impairments of Spatial Memory and N-methyl-d-aspartate Receptors and Their Postsynaptic Signaling Molecules in the Hippocampus of Developing Rats Induced by As, Pb, and Mn Mixture Exposure
Source: Brain Sci. 2023 Dec 14;13(12):1715. doi: 10.3390/brainsci13121715 (PMC10742016; doi:10.3390/brainsci13121715)

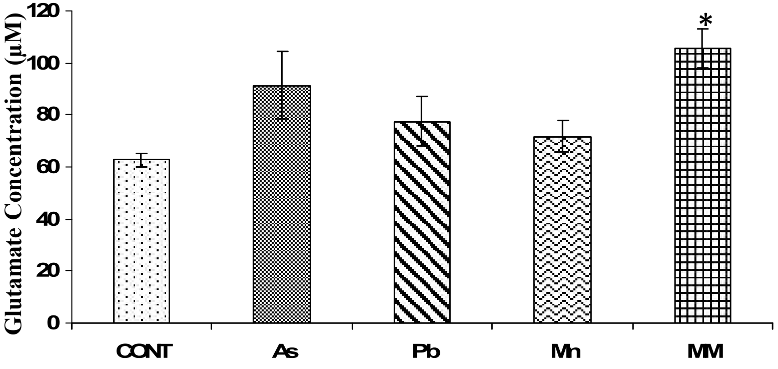

Suppl. Figure S1. Effect on glutamate concentration in hippocampus of rats following exposure (GD6-PD59) to As, Pb, Mn and their mixture on PD60. Values are the mean ± SEM of five animals in each group of both tests. Data have been analyzed by one-way analysis of variance followed by Newman–Keuls test. Significantly different $*p < 0.05$, as compared to controls.

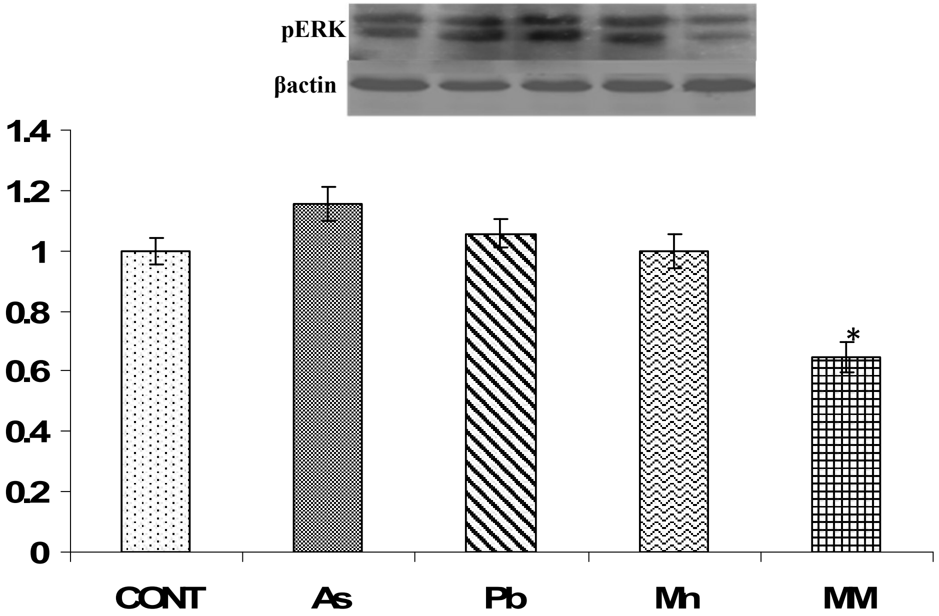

Suppl. Figure S2. Effect on expression pERk1/2 in hippocampus of rats following exposure (GD6-PD59) to As, Pb, Mn and their mixture on PD60. Values are the mean ± SEM of three animals in each group of both tests. Data have been analyzed by one-way analysis of variance followed by Newman–Keuls test. Significantly different $*p < 0.05$, as compared to controls.

Syngap

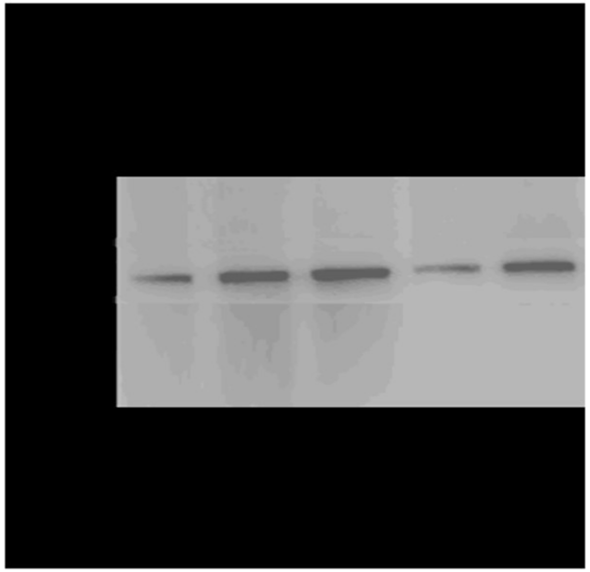

NR2A

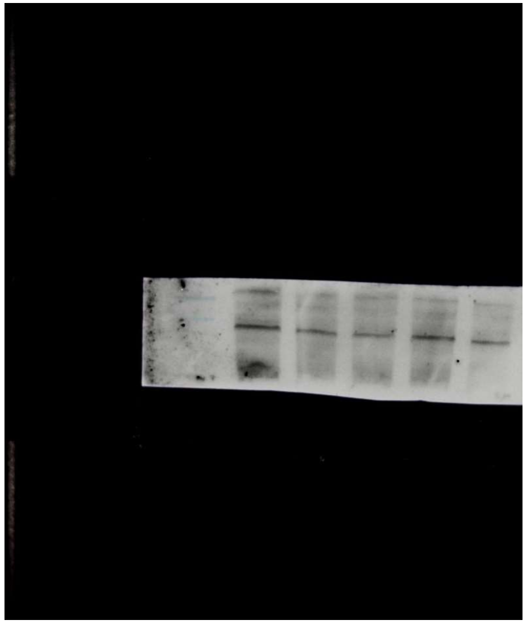

NR1

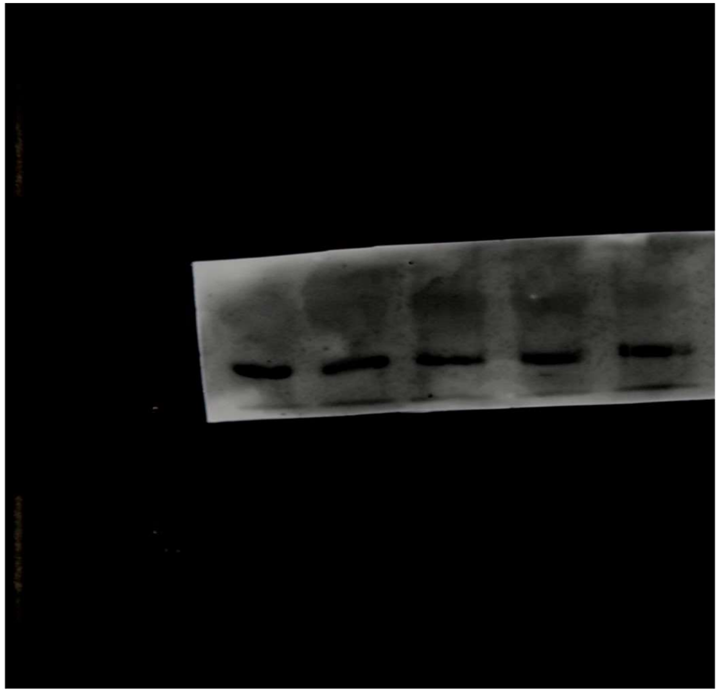

camkii

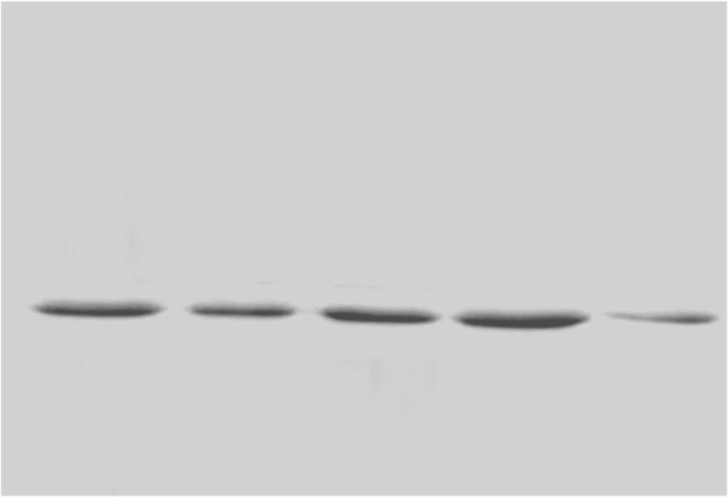

Beta actin

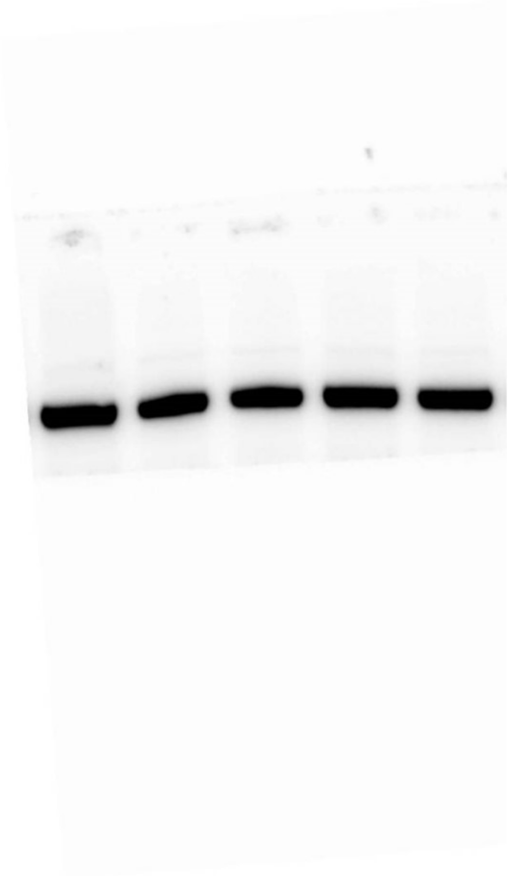

Supplement: Supplementary file 1 [file brainsci-13-01715-s001.zip › brainsci-2739618-supplementary.pdf]
